# Associations of C-Reactive Protein, Granulocytes and Granulocyte-to-Lymphocyte Ratio with Mortality from Breast Cancer in Non-Institutionalized American Women

**DOI:** 10.1371/journal.pone.0157482

**Published:** 2016-06-13

**Authors:** Wahyu Wulaningsih, Lars Holmberg, Lucie Abeler-Doner, Tony Ng, Sabine Rohrmann, Mieke Van Hemelrijck

**Affiliations:** 1 Cancer Epidemiology Group, Division of Cancer Studies, King’s College London, London, United Kingdom; 2 Division of Haematology/Oncology, Universitas Gadjah Mada, Yogyakarta, Indonesia; 3 Department of Surgical Sciences, Uppsala University, Uppsala, Sweden; 4 Regional Cancer Centre, Uppsala University, Uppsala, Sweden; 5 Department of Immunobiology, King’s College London, London, United Kingdom; 6 Richard Dimbleby Department of Cancer Research, Randall Division and Division of Cancer Studies, Kings College London, London, United Kingdom; 7 Department of Medical Oncology, University College London Cancer Institute, University College London, London, United Kingdom; 8 Institute of Social and Preventive Medicine, University of Zurich, Zurich, Switzerland; University of South Alabama Mitchell Cancer Institute, UNITED STATES

## Abstract

Inflammation may play a role in breast cancer, but evidence in the general population is lacking. We investigated the association between serum inflammatory markers (C-reactive protein (CRP), absolute granulocyte count (AGC) and granulocyte-to-lymphocyte (G/L) ratio) and breast cancer (BCa) mortality in American women while accounting for adiposity. From the Third National Health and Nutrition Examination Survey (NHANES III) we selected all women aged 20+ without any known history of cancer (n = 7,780). Multivariable Cox regression models were used to assess CRP, AGC and G/L ratio in relation to mortality from BCa, all cancer, cardiovascular disease and all causes. Stratification analyses by body mass index (BMI) and waist circumference were performed to investigate the effect of adiposity on this association. During a mean follow-up of 167 months, 44 women died from BCa. After adjustments for BMI and waist circumference, only G/L ratio was associated to risk of BCa death (e.g. HR: 2.35, 95% CI: 1.36–4.06 for the 3^rd^ compared to the 1^st^ tertile, P_trend_ = 0.01). Except for a borderline interaction between CRP categories and obesity by BMI, no statistically significant interaction between markers and categories of BMI or waist circumference was observed. All three markers were associated with mortality from cardiovascular disease and all causes. Our findings support a role of inflammation in BCa mortality which may involve mechanisms apart from obesity, and potential usefulness of GLR as a marker in assessing inflammation and cancer.

## Introduction

Chronic inflammation is thought to play a role in development and progression of cancer [1]. It may promote early tumourigenesis via pro-inflammatory cytokines and reactive oxygen species. In breast cancer (BCa) it is also suggested that inflammation activates the NF-κB signalling pathway, which may lead to development of more aggressive tumour subtypes and BCa progression through crosstalk with the oestrogen receptor [2].

C-reactive protein (CRP) is probably the most widely investigated marker of inflammation in the context of cancer detection and prognosis. CRP >10 mg/L is generally accepted as an indication of acute inflammation, while lower values have been found in low-grade chronic inflammation [3]. Higher levels of post-diagnosis CRP have been linked with worse survival in various malignancies, including BCa [4–6], suggesting the importance of inflammation in cancer progression. However, a consistent association between this marker and cancer risk has only been demonstrated for lung cancer and colorectal cancer [7,8]. For BCa, a meta-analysis including over 190,000 participants showed no risk difference in BCa incidence by CRP levels [9]. Furthermore, although it is thought that obesity may underlie the link between inflammation and breast cancer, the effect of obesity in this association is not well-defined [10,11].

Blood neutrophils, often measured as the absolute neutrophil count (ANC), are another inflammatory marker related to cancer prognosis [12]. Their prognostic value was however found to be limited in studies with long-term follow-up for cancer progression [13]. More recently, a ratio of neutrophil to lymphocyte counts in blood (neutrophil-to-lymphocyte ratio; NLR) was found as a predictor of both short- and long-term mortality in BCa patients, with higher values being associated with shorter survival [14,15]. Although total white blood count (WBC) is routinely used as an indicator of inflammation in clinic, the usefulness of these WBC components in risk prediction of BCa has not yet been investigated.

Using data from the Third National Health and Nutrition Examination Survey (NHANES III), the current study aims to investigate the link of CRP and NLR with BCa-specific death while accounting for adiposity. Granulocytes, which majorly consist of neutrophils [16], were used as a surrogate for neutrophils as the latter were only available in a small number of participants in the survey.

## Methods

### Study population

The National Center for Health Statistics (NCHS) conducted NHANES III between 1988 and 1994 and designed it as a multistage stratified, clustered probability sample of the US civilian non-institutionalized population who was at least two months old. All subjects participated in an interview conducted at home and an extensive physical examination, which included a blood sample taken in a mobile examination center [17]. Despite a cross-sectional design, the NHANES III dataset was linked with the National Death Index (NDI) maintained by NCHS through December 31, 2006 [18], allowing the use of the dataset as a prospective cohort. From this population, we included a total of 7,780 females aged 20 and over with baseline measurements of serum CRP, differential WBC, and eligible mortality follow-up information. No participants reported a history of cancer at baseline. The protocols for the conduct of NHANES III were approved by the Institutional Review Board of the NCHS, Centers for Disease Control and Prevention. Written informed consent was obtained from all participants.

### Exposure measurements

CRP was measured with an automated Behring Nephelometer Analyzer System (Behring Diagnostics, Inc, Somerville, NJ) [19]. Coefficients of variation ranged from 3.2% to 16.0% throughout data collection. Tests were repeated for specimens with results of > 10 mg/L. Because levels of CRP below 2.2 mg/L were undetectable in the NHANES III, we used clinical cut-offs as previously described [20]: undetectable (<2.2 mg/L), intermediate (2.2–10 mg/L) and clinically raised (≥ 10 mg/L). Differential WBC were measured with automated Coulter Counter S-PLUS JR Hematology Analyzer (Coulter Electronics, Hialeah, FL) [19]. The maximum coefficients of variation were 5% for absolute granulocyte count and 5% for absolute lymphocyte count. The G/L ratio was calculated as a ratio of absolute granulocyte to lymphocyte count.

### Covariates

Information on age, race/ethnicity, cigarette smoking, alcohol consumption, physical activity, aspirin use, BCa risk factors, history of diabetes, cardiovascular disease and cancer was collected during the interview. Race and ethnicity were combined into four racial/ethnic groups: non-Hispanic white, non-Hispanic black, Mexican American, and other. Participants were classified as never, former, and current smokers (< 20, 20–40, ≥ 40 cigarettes per day), based on the self-reported smoking habits. Frequency of alcohol consumption was measured by a food frequency questionnaire and categorised by times per week. Vigorous physical activity (yes, no) was defined as participating three or more times per week in the following activities: jogging or running; swimming or aerobics (age <65 years); biking, dancing, gardening, and calisthenics (≥65 years); and walking and lifting weights (≥80 years). Aspirin use (yes, no) was assessed based on responses to the question “How often did you take aspirin during the past month?”. Collected information on BCa risk factors included self-reported menopausal status, parity, age of menarche, age at first childbirth, history of breastfeeding, use of contraceptive pills and hormone replacement therapy. Menopausal status was assessed by the following self-reported criteria: premenopause: last period was 12 and postmenopause: last period >12 months. Women with bilateral oophorectomy and/or hysterectomy were included in the menopause group. Parity was grouped based on the number of live births: 0, 1, and 2 or more. History of breastfeeding was self-reported among parous women. Body measurements were taken during the examination [17] which included body mass index (BMI) calculated from participants weight and height, and waist circumference.

### Outcomes

The main outcome of the present study was BCa death, however, we also collected data for all-cause mortality as well as cardiovascular and cancer-specific death as competing outcomes. Underlying cause of death was based on ICD-9 codes through 1998 and on ICD-10 codes for deaths occurring after 1998. In order to adjust for changes between the two coding systems, final cause of deaths occurring prior to 1999 were re-coded into comparable ICD-10-based underlying cause of death groups [18]. Follow-up time was calculated from interview date/examination date until date of death or end of study (31 December, 2006), whichever came first [18].

### Statistical analysis

Sampling weights for NHANES III were used to account for sampling variability and to adjust for differential probability of selection of persons [17]. Multivariable Cox proportional hazards regression was used to assess risk of death by clinical cut-offs of CRP, as well as log-transformed values and tertiles of detectable CRP, AGC, and G/L ratio. A test for trend was conducted by using assignment to categories as an ordinal scale. In the multivariable model, we performed further adjustment for cigarette smoking, alcohol consumption, vigorous physical activity, use of aspirin. Additional adjustment for BCa risk factors was performed when assessing BCa death. Due to the large number of missing data (> 10%) of other risk factors, only menopausal status, age at menarche and use of contraceptive pills were included in the final analyses, and missing values for these variables were coded as a separate category and included in the analysis. To account for potential confounding by obesity [10], we repeated our analyses while adjusting the models for BMI and WC. Given their potential overlap, we conducted a test for collinearity with variance inflation factor (VIF) for BMI and waist circumference as predictors of BCa death. VIF obtained was less than 2, indicating a lack of severe multi-collinearity [21]. Therefore, we retained both BMI and waist circumference in the model. Sensitivity analyses for BCa death was performed by excluding those with follow-up time less than 3 years (N = 957). Finally, to evaluate effect modification, we performed stratified analyses by obesity using BMI 30 kg/m^2^ as a cut-off and the median of waist circumference. We also performed a test for multiplicative interaction by including an interaction term between categories of inflammatory markers and obesity status, categories of waist circumference or menopausal status in the model. Due to small number of BCa deaths in these stratified analyses, only log-transformed values and trends from analyses with categories were displayed. Cumulative incidence functions were created for death from different causes, and for BCa death by clinical cut-offs of CRP and tertiles of AGC and G/L ratio. We performed Gray’s Test for Equality of Cumulative Incidence Functions to assess between-groups differences in the presence of competing risks. All analyses were conducted with SAS release 9.4 (SAS Institute, Cary, NC) and R version 3.1.0 (R Foundation for Statistical Computing, Vienna, Austria).

## Results

During a mean follow-up time of 167 months, a total of 1,676 women died: 307 of cancer, including 44 who died of BCa, and 779 of cardiovascular disease. Baseline characteristics of study participants are shown in Table 1.

**Table 1:** Characteristics of study participants by status at the end of follow-up.

	Weighted mean (SD)/ N (weighted %)				
	Breast cancer death (n = 44)	All cancer death (n = 307)	Cardiovascular death (n = 779)	All death (n = 1,676)	Alive (n = 6,104)
**Age** (years)					
Mean (SD)	48.93 (3.83)	60.13 (1.41)	71.87 (0.86)	67.60 (0.74)	40.81 (0.43)
**Mean follow-up** (months)					
Mean (SD)	110.57 (11.12)	101.62 (4.40)	97.42 (2.37)	101.67 (1.95)	179.65 (2.88)
**Race—Ethnicity**					
Non-Hispanic white	16 (71.45)	163 (81.99)	480 (83.97)	978 (82.26)	2255 (74.60)
Non-Hispanic black	17 (14.91)	84 (11.34)	165 (10.66)	389 (11.21)	1792 (11.29)
Mexican American	9 (3.26)	51 (2.53)	113 (2.11)	261 (2.34)	1758 (5.26)
Other	2 (10.38)	9 (4.13)	21 (3.27)	48 (4.19)	299 (8.85)
**Alcohol consumption**					
Never	28 (57.90)	205 (62.20)	641 (75.67)	1312 (71.51)	3603 (49.70)
Up to 6 times a week	10 (29.97)	77 (19.85)	107 (13.01)	280 (14.47)	2257 (22.24)
Daily or more	6 (12.12)	25 (7.79)	31 (6.71)	84 (6.69)	244 (5.14)
**Cigarette smoking**					
Never	23 (58.84)	140 (38.05)	515 (58.36)	1001 (50.29)	3825 (56.31)
Former	5 (10.31)	70 (27.61)	165 (23.97)	362 (25.43)	949 (18.60)
Current	16 (30.85)	97 (34.34)	99 (17.67)	313 (24.28)	1330 (25.09)
**Vigorous Physical activity**	3 (11.70)	53 (19.83)	179 (27.40)	353 (24.00)	367 (6.21)
**Waist circumference** (cm) ^1^	99.30 (4.59)	98.93 (1.54)	95.08 (0.88)	94.94 (0.53)	87.61 (0.43)
**BMI** (kg/m^2^)	29.66 (1.71)	29.90 (0.88)	27.23 (0.32)	27.39 (0.15)	26.32 (0.20)
**Menopausal status**^2^					
Premenopause	17 (51.51)	51 (16.88)	35 (5.90)	138 (10.46)	3961 (64.14)
Postmenopause	26 (48.49)	240 (79.28)	628 (92.96)	1347 (87.89)	1974 (29.85)
**Age at menarche**^**3**^ (years)					
Mean (SD)	12.94 (0.50)	12.69 (0.11)	13.09 (0.07)	13.00 (0.07)	12.76 (0.04)
**Age at first childbirth**^**4**^ (years)					
Mean (SD)	22.14 (0.76)	22.50 (0.36)	22.77 (0.22)	22.65 (0.18)	22.24 (0.13)
**History of breastfeeding**^5^	19 (40.24)	157 (58.68)	393 (66.38)	845 (62.01)	394 (53.75)
**Parity**^6^					
0	3 (12.07)	16 (6.17)	26 (4.53)	53 (3.85)	319 (8.37)
1	6 (17.07)	37 (14.78)	113 (18.47)	223 (16.55)	1001 (20.43)
2+	29 (70.86)	208 (79.05)	441 (77.00)	1016 (79.60)	3806 (71.20)
**History of oral contraception**^7^	22 (72.90)	86 (39.61)	68 (13.35)	226 (35.10)	3842 (45.92)
**Hormone replacement therapy**^**8**^	3 (14.62)	71 (41.03)	159 (34.32)	345 (21.28)	844 (78.72)
**Aspirin use**	17 (46.44)	114 (39.51)	301 (45.32)	608 (40.50)	1759 (33.88)
**CRP (mg/L)**					
Undetectable (< 2.2)	22 (56.9)	177 (57.25)	432 (54.65)	909 (54.75)	3773 (67.98)
Intermediate (2.2–10)	13 (24.22)	84 (25.46)	225 (27.38)	492 (26.91)	1639 (23.43)
Clinically raised (≥ 10)	9 (18.78)	46 (17.29)	122 (17.97)	275 (18.34)	692 (8.60)
**AGC (10**^**3**^ **cells/L)**					
Mean (SE)	5.39 (0.53)	4.82 (0.18)	4.85 (0.09)	4.85 (0.07)	4.56 (0.05)
**G/L ratio**					
Mean (SE)	2.61 (0.27)	2.29 (0.11)	2.45 (0.05)	2.38 (0.04)	2.11 (0.03)

Information available in ^1^7618, ^2^7420, ^3^7285, ^4^6031, ^5^6044, ^6^6418, ^7^7410 and ^8^3422 women.

### CRP, AGC, G/L ratio and BCa death

**Fig 1:**
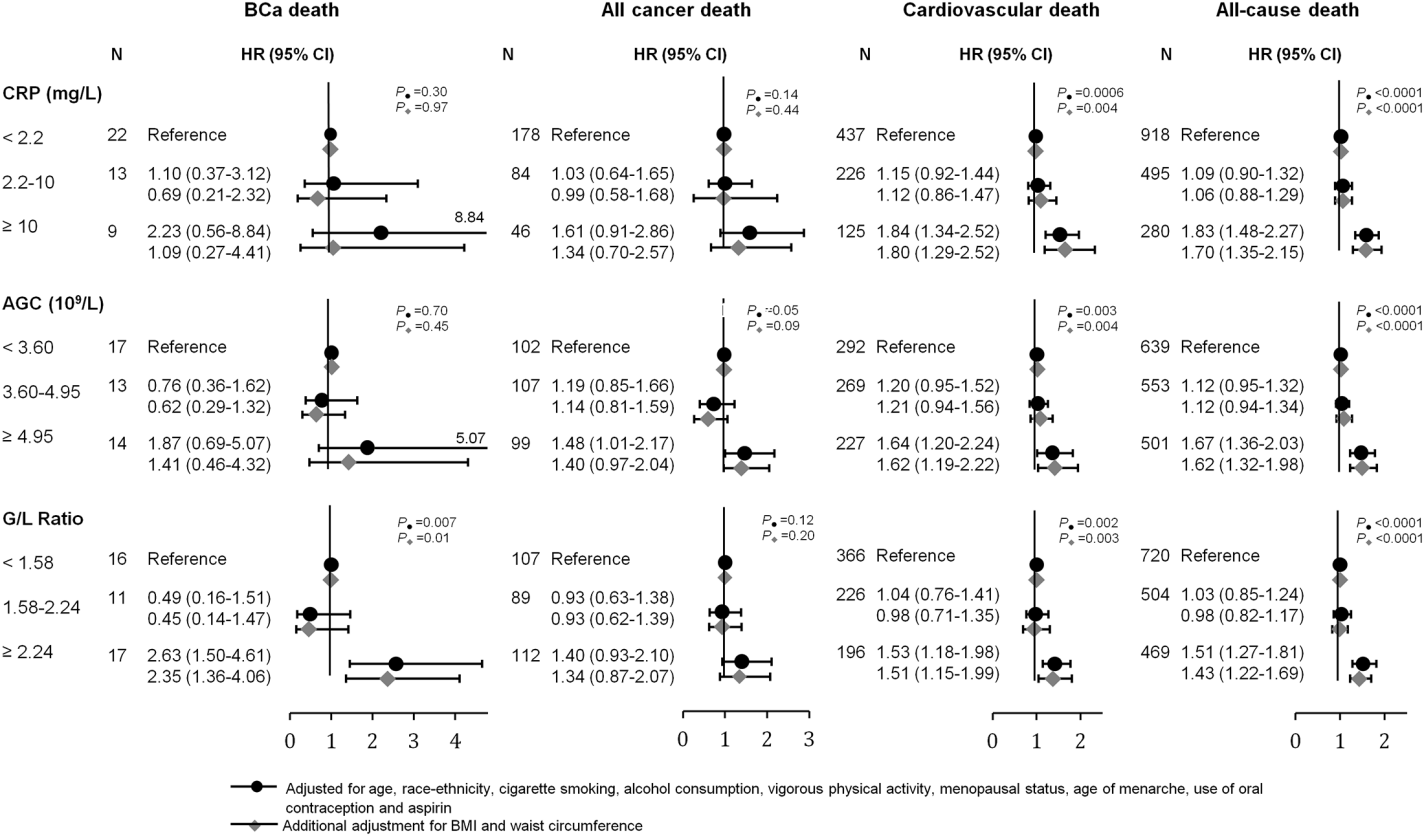
Hazard ratios (HR) and 95% confidence intervals (95% CI) for risk of death from BCa, overall cancer, cardiovascular disease and all causes by clinical cut-offs of CRP and tertiles of absolute granulocyte count and G/L ratio. All models were adjusted for age, race-ethnicity, cigarette smoking, alcohol consumption, vigorous physical activity, menopausal status, age of menarche, use of oral contraception and aspirin.

When using the clinical cut-off of CRP, we did not observe any statistically significant association between CRP and risk of BCa death. However, among those women with detectable CRP levels, higher CRP was associated with an increased risk of BCa death (HR: 1.82 (95% CI: 1.02–3.25) for every log increase). We also found continuous AGC to be positively associated with BCa death (HR: 3.96 (95% CI: 1.10–14.12) for every log increase in AGC), although this was not observed with tertiles of AGC (Fig 1). A positive association with BCa death was also found for both the log-transformed G/L ratio as well as the tertiles of G/L (e.g. HR: 4.28 (95% CI: 1.88–9.74) for every log increase in G/L ratio). The results were similar when we excluded women with less than three years of follow-up (results not shown).

### Effects of obesity and menopausal status

When we performed an additional adjustment for BMI, the observed associations between log-transformed values of detectable CRP and AGC with BCa death disappeared (results not shown). Lack of associations were also found when using CRP cut-offs and AGC tertiles (Fig 1), whereas G/L ratio remained statistically significantly associated with risk of BCa death (e.g HR: 2.35 (95% CI: 1.36–4.06) for the highest compared to lowest tertile of G/L ratio in the BMI-adjusted model; P_trend_ = 0.01). Findings were similar in the sensitivity analyses. For instance, the HR for BCa death was 4.02 (95%: 1.64–9.85) for continuous G/L ratio when excluding women with less than 3 years of follow-up (results not shown in tables). When stratifying by overweight status and categories of waist circumference, we only observed a positive trend between continuous log-transformed values of G/L ratio and risk of BCa death in women with waist circumference above the median (Table 2), with hazard ratios of 7.20 (95% CI: 1.29–40.25). However, there was no statistically significant interaction between the three markers and obesity status apart from a borderline interaction between CRP and obesity by BMI (P_interaction_ = 0.05). In another stratified analyses based on menopausal status, no associations were found with BCa death for premenopausal or postmenopausal women separately. A lack of statistically significant interaction was also found between all three inflammatory markers and menopausal status when assessing BCa death (P_interaction_ ≥0.05).

**Table 2:** Hazard ratios (HR) and 95% confidence intervals (95% CI) for risk of death due to BCa, overall cancer, cardiovascular disease and all causes by continuous levels of clinically detectable CRP, absolute granulocyte count and G/L ratio, and trends from categories of markers, stratified by overweight status and median WC. All models were adjusted for age, race-ethnicity, cigarette smoking, alcohol consumption, vigorous physical activity, menopausal status, age of menarche, use of oral contraception and aspirin.

	N	CRP (mg/L)			AGC (10^3^/L)			G/L ratio		
		HR (95% CI) per log increase^1^	P_tremd_^2^	P_interaction_	HR (95% CI) per log increase	P_tremd_^2^	P_interaction_	HR (95% CI) per log increase	P_tremd_^2^	P_interaction_
**Stratification by BMI (kg/m**^**2**^**)**										
**BMI < 30** (N = 5462)										
Breast cancer death	21	1.94 (0.51–7.29)	0.39		5.48 (0.45–66.27)	0.52		6.51 (0.94–20.03)	0.38	
All cancer death	208	1.13 (0.76–1.67)	0.27		1.63 (1.01–2.63)	0.03		1.11 (0.68–1.80)	0.24	
Cardiovascular death	569	1.45 (1.15–1.82)	0.12		2.22 (1.39–3.56)	0.007		1.99 (1.49–2.65)	0.003	
All death	1207	1.49 (1.27–1.76)	0.002		2.00 (1.52–2.66)	<0.0001		1.62 (1.32–2.00)	<0.0001	
**BMI ≥ 30** (N = 2318)										
Breast cancer death	23	1.40 (0.52–3.77)	0.76	0.05	2.85 (0.31–25.92)	0.58	0.73	6.51 (0.97–43.74)	0.13	0.93
All cancer death	99	1.26 (0.65–2.47)	0.43	0.15	1.97 (0.69–5.61)	0.58	0.59	3.11 (0.87–11.17)	0.16	0.41
Cardiovascular death	210	1.29 (0.77–2.17)	0.43	0.79	1.66 (0.73–3.76)	0.51	0.37	1.46 (0.76–2.83)	0.18	0.61
All death	469	1.43 (1.07–1.91)	0.24	0.43	1.89 (1.16–3.08)	0.05	0.53	1.85 (1.12–3.06)	0.02	0.82
**Stratification by waist circumference (cm)**										
**Waist circumference < 90.10** (N = 3615)										
Breast cancer death	11	2.30 (0.39–13.43)	0.78		1.01 (0.12–9.56)	0.82		1.51 (0.25–9.10)	0.86	
All cancer death	108	0.88 (0.37–2.10)	0.93		1.38 (0.70–2.69)	0.25		1.37 (0.70–2.69)	0.50	
Cardiovascular death	230	2.36 (1.64–3.40)	0.003		2.07 (1.06–4.03)	0.03		1.96 (1.24–3.08)	0.05	
All death	514	1.72 (1.26–2.36)	0.03		1.60 (1.12–2.30)	0.01		1.48 (1.15–1.91)	0.03	
**Waist circumference ≥ 90.10** (N = 4003)										
Breast cancer death	33	1.81 (0.98–3.35)	0.41	0.22	5.16 (0.59–44.96)	0.32	0.55	7.20 (1.29–40.25)	0.07	0.29
All cancer death	190	1.22 (0.74–2.02)	0.25	0.67	1.70 (0.86–3.35)	0.30	0.97	1.51 (0.60–3.77)	0.21	0.79
Cardiovascular death	465	1.20 (0.82–1.76)	0.07	0.11	2.06 (1.35–3.15)	0.81	0.90	1.85 (1.28–2.66)	0.004	0.96
All death	1030	1.40 (1.15–1.68)	0.007	0.49	2.13 (1.59–2.86)	0.77	0.69	1.82 (1.32–2.51)	0.0003	0.30

^1^ Clinically detectable levels of CRP were used as a continuous variable (≥ 2.2 mg/L)

^2^ P_trend_ from regression models using clinical categories of CRP and tertiles of AGC and G/L ratio

### CRP, AGC, G/L ratio and other causes of death

**Fig 2:**
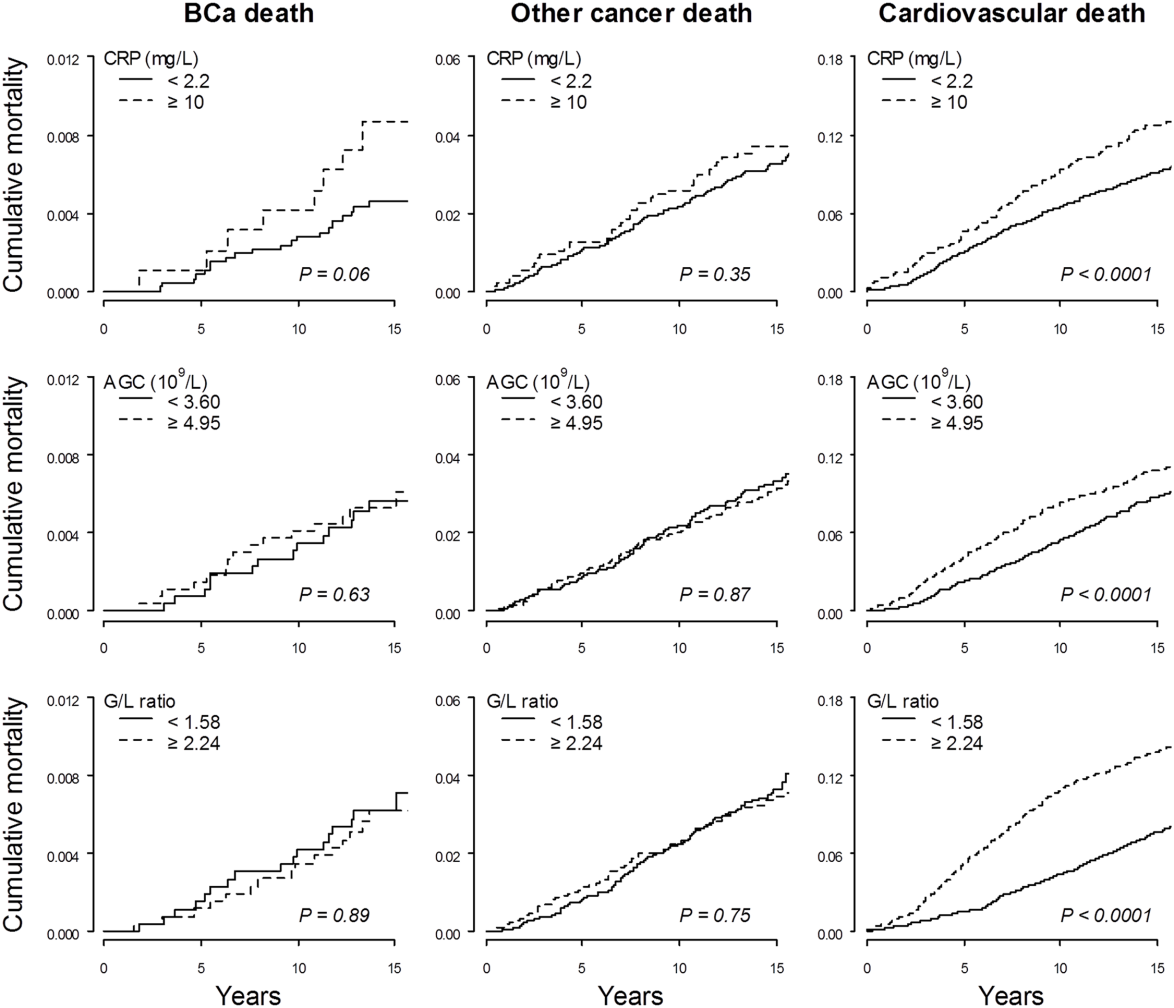
Cumulative mortality from BCa, other cancer and cardiovascular disease by the highest and lowest categories of CRP, AGC and G/L ratio. Gray’s test for equality of cumulative incidence functions was performed in presence of competing risks.

Only AGC was associated to risk of overall cancer death, e.g. HR: 1.48 (95% CI: 1.01–2.17) for the 3^rd^ compared to the 1^st^ tertile (Fig 1). The risks of dying from cardiovascular disease and all causes were consistently higher with increased CRP, AGC and G/L ratio (Fig 1). Interestingly, no effect modification by obesity status by BMI and waist circumference was observed for the association between any markers and risk of other causes of death apart from BCa (Table 2). Fig 2 shows the distribution of cumulative mortality from BCa, other cancer, cardiovascular disease and other causes for women in the highest and lowest categories of CRP, AGC, and G/L ratio. When accounting for other causes of death as competing risks, we found higher BCa mortality rates over time in women in the top CRP and GLR categories compared to the lowest, but no statistically significance difference was observed (Gray’s test: P ≥0.05). Similar but stronger associations for the three inflammatory markers were found for cardiovascular death (Fig 2).

## Discussion

In the present study, the G/L ratio was shown to be linked to BCa death even after taking into account adiposity. Although a borderline interaction between CRP and obesity by BMI was observed, no statistically significant interaction between any inflammatory markers and obesity status or menopausal status was found. Additionally, levels of CRP, AGC and G/L ratio were statistically significantly associated with risk of cardiovascular and all-cause death. No association was observed between the studied inflammatory markers and risk of all-cancer death after obesity was taken into account.

Observations of leukocytes infiltration in tumour tissue were among the earliest evidence indicating a link between inflammation and cancer [22]. The inflammatory milieu has been recognised as an important part of cancer microenvironment, but in contrast to typical inflammatory responses, it is characterized by defective immune effectors including alternatively-activated macrophages [23], dysfunctional dendritic cells (DC) [24], mast cells [25] and lymphocytes [26]. It is thought that these impaired immune responses in cancer are driven by a subpopulation of myeloid-derived cells with immunosuppressive properties, comprising macrophages, granulocytes, and DCs at varying stages of differentiation and expressing specific markers including CD11b and Gr-1 [27]. Corroborating this hypothesis, a suppression of cytotoxic T cells activity by CD11b+ granulocytes and macrophages in animal models of cancer has been reported [28], which is thought to underlie the immune tolerance to cancer. Correspondingly, infiltrates of T cells triggered by tumour-associated antigens are linked to better survival in cancer patients [29], whereas tumour-associated macrophages have been associated to metastasis and worse prognosis [30].

How systemic inflammation fits into this relationship between inflammation and cancer remains elusive, since tumour-infiltrating immune cells harbour different characteristics from their equivalents in circulation [22]. Nevertheless, similar to contrasting effects between infiltrates of granulocytes and lymphocytes, circulating granulocytes have been reported to inhibit the cytotoxic effect of lymphocytes [31], implying the potential usefulness of systemic inflammatory markers in relation to cancer. However, underlying factors such as obesity [32] and infection [33] might be triggers of activation of these immune cells and cancer initiation.

In the context of prognosis, increased pre-treatment CRP levels in BCa patients have been linked with decreased progression-free and overall survival, with a hazard ratio for disease progression of 1.65 (95% CI: 1.26–2.15) for women in the highest tertile of CRP compared to the lowest [34]. The association between CRP and risk of being diagnosed with BCa is less clear [9,35]. In 9,605 women included in the Copenhagen City Heart Study [36], no association was reported between baseline CRP and risk of developing BCa (HR: 0.70 (95% CI, 0.40 to 1.40) for CRP more than 3 versus less than 1 mg/L) when adjusting for lifestyle factors including BMI. A lack of association between CRP and BCa incidence was also reported in a recent study of 155,179 Swedish women [37]. In contrast, a nested case-control study in France showed a positive association between CRP and postmenopausal BCa among overweight and obese women or those with waist circumference ≥ 80 cm, with a significant interaction between CRP and BMI [38]. Similar to the null finding in the Danish study [36], we found lack of associations between CRP and risk of BCa death after adjustment for BMI and waist circumference. It is of note, however, that we did not have information on BCa incidence. Therefore, the use of BCa death as a surrogate endpoint may have underestimated the association between inflammatory markers and BCa and is not fully comparable to findings from studies where diagnosis of BCa is available. With respect to adiposity, a positive association between clinically detectable CRP (≥2.2 mg/L) and risk of BCa death was observed for overweight and obese women and those with waist circumference above median. Nevertheless, no statistically significant interaction was found, although a borderline interaction was found with obesity as defined by BMI. Therefore, our findings did not support any major effect of adiposity on the association between CRP and BCa death. =

Neutrophils are the most abundant granulocytes in the circulation [16], and ANC is routinely used as marker in monitoring chemotherapy-induced myelosuppression [39]. With regards to BCa diagnosis, however, there is a lack of evidence assessing the potential significance of this marker. In the present study, we found no association between AGC and risk of BCa death after adjustment for BMI. No participants reported a history of cancer diagnosis at baseline, so that alterations of neutrophil levels by cancer or its treatment were unlikely to affect the results of this study.

Similar to NLR [40], a high G/L ratio has been reported to be linked to worse prognosis in colorectal cancer in studies on cancer prognosis [41], but its importance in relation to BCa incidence is not widely investigated yet. In our study, the G/L ratio was a strong predictor of BCa death and unlike AGC and CRP, it was not affected by BMI and waist circumference adjustments. Furthermore, this association was robust after exclusion of women with less than three years of follow up. In the stratified analysis, there was a weak indication that the link between the G/L ratio and BCa death was affected by menopausal status, but the small sample size may have driven the lack of statistical power. Stratification by overweight status showed a stronger associations between G/L ratio and BCa death in overweight and obese women and those with waist circumference above median, which may support the proposed obesity-inflammation-cancer axis in breast carcinogenesis [42]. Nevertheless, the lack of significant interaction may point towards the importance of alternative inflammatory pathways.

Finally, the strong association between markers of inflammation and death from cardiovascular disease and all causes highlight the importance of competing risks when analysing inflammatory markers in relation to time to cancer death. Cardiovascular disease is the most frequent cause of death [43] and may result in a competing risk situation when assessing BCa death. Cumulative mortality rates of cardiovascular death were markedly higher in women with elevated levels of CRP, AGC, and G/L ratio. Therefore, any woman with high baseline levels of inflammatory markers who are at risk of dying from BCa was more likely to die first from cardiovascular disease before experiencing fatal BCa [44], and this may explain the weaker effects of serum CRP and G/L ratio on cumulative mortality from BCa. However, we observed similar but non-significant trends of CRP and G/L ratio with cumulative BCa death after taking competing risk into account.

The strength of this study is its generalisability following the use of nationally representative data of the US population. We were able to adjust for potential confounders and stratify by overweight and menopausal status in women. A limitation of this study is that it relied on a single measurement, so that it may be prone to measurement error and within-person variation. As inflammation arises following cancer [1], effects of reverse causation may remain even after excluding those with < 3 years of follow-up. Additionally, the laboratory methods used for CRP measurement at the time the NHANES III was conducted were unable to perform a high sensitivity assay of this marker. Nevertheless, serum CRP in the NHANES III population was reported to be associated to CRP-related genetic variation [45], justifying the usefulness of this marker despite its limitation in detecting low levels of CRP. In stratification analyses, we only used continuous values of markers given the small number of events, and for CRP, this excluded half (50%) of BCa cases with undetectable levels. This exclusion may limit the generalisability to our findings for this particular analysis to those with mild to clinically evident systemic inflammation. The small number of BCa deaths was also a major limitation of our study and may have resulted in imprecision. Additionally, no history of breast cancer diagnosis was available. Finally, BCa is a disease with high survivorship. Using BCa mortality due to a lack of information on cancer incidence in our study may have underestimated the association between inflammation and BCa. Consequently, our results may have implied a role of inflammation in more severe BCa, which may be represented by women dying specifically from BCa. Further investigations including cancer incidence and prognostic indicators are needed to clarify this association.

## Conclusion

Our findings support a role of inflammation in breast cancer. Although we found, the association between serum inflammatory markers and BCa to be more profound with excess adiposity, no statistically significant interaction between any of the three inflammatory markers investigated and BMI or waist circumference as indicators of obesity. This may imply alteran the need for future mechanistic studies investigating underlying biological pathways linking inflammation and breast cancer beyond obesity. Additionally, we demonstrated that the G/L ratio, an alternative marker for inflammation, may be useful in assessing risk of BCa mortality in women without known history of cancer. However, weaker associations were observed in presence of competing risks, and therefore addressing other causes of death is important in assessing inflammatory markers in relation to BCa death.
