# Correction: Associations of C-Reactive Protein, Granulocytes and Granulocyte-to-Lymphocyte Ratio with Mortality from Breast Cancer in Non-Institutionalized American Women

**DOI:** 10.1371/journal.pone.0161418

**Published:** 2016-08-11

**Authors:** Wahyu Wulaningsih, Lars Holmberg, Lucie Abeler-Dörner, Tony Ng, Sabine Rohrmann, Mieke Van Hemelrijck

The third author’s name is spelled incorrectly. The correct name is: Lucie Abeler-Dörner. The correct citation is: Wulaningsih W, Holmberg L, Abeler-Dörner L, Ng T, Rohrmann S, Van Hemelrijck M (2016) Associations of C-Reactive Protein, Granulocytes and Granulocyte-to-Lymphocyte Ratio with Mortality from Breast Cancer in Non-Institutionalized American Women. PLoS ONE 11(6): e0157482. doi:10.1371/journal.pone.0157482
